# Perceptions of a comprehensive telehealth intervention in patients with persistently poor type 2 diabetes control

**DOI:** 10.1017/cts.2025.10082

**Published:** 2025-06-25

**Authors:** Jashalynn German, Madeleine R. Eldridge, Lucy Esteve, Anastasia-Stefania Alexopoulos, Connor Drake, Allison Lewinski, Hayden B. Bosworth, David Edelman, Karen Steinhauser, Matthew J. Crowley

**Affiliations:** 1 Department of Medicine, Duke University, Durham, USA; 2 Durham Veterans Affairs Center of Innovation to Accelerate Discovery and Practice Transformation (ADAPT), Durham, USA; 3 Department of Population Health Sciences, Duke University School of Medicine, Durham, USA; 4 Duke University School of Nursing, Durham, USA

**Keywords:** Type 2 diabetes, telehealth, patient perspectives, staff perspectives, qualitative research

## Abstract

**Introduction:**

To understand participant perspectives on an effective, practical, comprehensive telehealth intervention for persistently poorly controlled diabetes mellitus and examine how its components contributed to improved outcomes, with the goal of informing broader telehealth-based diabetes management strategies.

**Methods:**

We conducted semi-structured interviews of a purposive sample of patients and staff in the comprehensive telehealth arm of the Practical Telehealth to Improve Control and Engagement for Patients with Clinic-Refractory Diabetes Mellitus study. Using the lens of patient engagement, we applied directed content analysis to categorize themes across the five components of the intervention.

**Results:**

The purposive sample included 19 patients (79% male, 53% Black, varying levels of intervention engagement) and 8 staff. The telemonitoring component was associated with encouragement and motivation among patients; staff found satisfaction in providing metrics of success for participants. For the self-management component, patients saw staff as helpful with problem-solving; staff felt patients were receptive to education. Medication management supported medication adherence and optimization and was acceptable to patients. Diet/activity support motivated behavioral changes among patients. Staff felt that depression support allowed for responsiveness to medical and behavioral factors influencing self-management. Identified areas for improvement included staff time constraints, patient difficulties with taking and transmitting data, and challenges with patient adherence among those with mental health conditions.

**Conclusion:**

Findings from this study provide insights that may inform the design, implementation, and scalability of comprehensive telehealth models for diabetes management across diverse healthcare settings.

## Introduction

The rising prevalence of type 2 diabetes mellitus (T2 DM) and associated complications imposes a significant burden on patients and healthcare systems [1]. More than 37 million Americans have T2 DM, with annual cost of care exceeding $400 billion [2,3]. Among patients with T2 DM who received regular clinic-based care (defined as at least one primary care visit and two hemoglobin A1c values over one year), approximately 12% have persistently poorly controlled diabetes mellitus (PPDM). This subgroup of patients is at particularly high risk of diabetes-related morbidity and mortality [3–6]. Factors that may increase the likelihood of PPDM include clinical inertia, unavailable blood glucose data, suboptimal lifestyle modification, complex medication regimens/nonadherence, and competing comorbidities [7–14].

The effectiveness of telehealth interventions in improving glycemic control is increasingly recognized [15–19]. Telehealth may be a useful strategy for improving outcomes in PPDM, particularly through comprehensive telehealth interventions addressing multiple factors underlying PPDM. Practical Telehealth to Improve Control and Engagement for Patients with Clinic-Refractory Diabetes Mellitus (PRACTICE-DM, NCT03520413), a recently completed, active-comparator randomized clinical trial investigated the effectiveness of a comprehensive telehealth intervention to improve glycemic control in PPDM by leveraging existing clinical infrastructure within the Veteran Health Administration (VHA) system [4]. This comprehensive telehealth intervention consisted of five components: telemonitoring, self-management support, diet/activity support, medication management, and depression support, effectively improved hemoglobin A1c (HbA1c) levels, diabetes distress, self-efficacy, and self-care [20]. The randomized trial findings revealed that patients randomized to receive the comprehensive telehealth approach (*n* = 101) experienced better glycemic control than the standard telehealth active-comparator approach (*n* = 99) with HbA1c reductions of 1.59% and 0.98%, respectively, over a 12-month period (*P* = .02). However, little is known about how the intervention components, or comprehensive telehealth interventions more broadly, contribute to these positive outcomes in patients with PPDM.

The effectiveness of telehealth interventions in improving glycemic control is increasingly recognized, yet less is known about the factors driving these improvements. To understand the contributions of individual intervention components and identify opportunities for refinement to amplify its benefit on diabetes-related outcomes, we conducted semi-structured interviews with patient and staff participants. These interviews explored the underlying processes behind improved diabetes control. This work provides insights that can inform the design, implementation, and scalability of comprehensive telehealth models for diabetes management in diverse healthcare settings.

## Methods

### Overview

The PRACTICE-DM trial (NCT03520413) [20,21], approved by the Durham and Richmond VA Medical Center Institutional Review Boards, enrolled patients with PPDM (defined as ≥2 HbA1c values of ≥8.5%; without readings <8.5%; and ≥1 appointment with VHA primary care or endocrinology during the prior year) into a two-site, 12-month randomized trial. Participants were assigned to either standard VHA Home Telehealth (HT) care coordination and telemonitoring (daily automatic transmission of HT-issued glucometer data for compilation and provider review with communication for alarm values or other acute issues as needed) or a comprehensive telehealth intervention comprising five components: telemonitoring, self-management support (via a script-guided module-based approach covering topics including self-monitoring blood glucose, self-management of hypoglycemia, and insulin management (Appendix), diet/activity support, medication management (provided by physicians, clinical pharmacists, or nurse practitioners), and depression support (provided in conjunction with a study psychiatrist). The comprehensive telehealth intervention design was guided by a theoretical framework based on Cumulative Complexity Model, which indicates that patient engagement with interventions and subsequent outcomes are influenced by the balance of patient’s demands and the capacity to meet demand [21,22]. Both study arms were delivered using existing VHA HT staffing and infrastructure.

### Patient and staff interviews

At the end of the 12-month randomized trial, staff and patient participants in the comprehensive telehealth intervention arm were invited to complete semi-structured interviews about their study participation. Interviews were conducted by a methodological specialist with interviewing experience (ME) who is not a medical practitioner. A purposive sampling strategy was used to gather staff and patient participant perspectives from both site locations and patient participants of varying levels of engagement. Patient engagement was assessed based on the number of calls completed with HT nurses (1–9 calls = low engagement, 10–18 calls = moderate engagement, and ≥19 calls = high engagement). Eight staff (3 HT nurses, 5 medication managers) and 20 patients consented to audio recorded, semi-structured interviews; however, one patient was later excluded due to considerations surrounding their mental health, which led to interview responses that were deemed unreliable. Recruitment and data collection of 20 patients represented approximately 20% of the comprehensive intervention arm of the PRACTICE-DM study. This sample size was informed by anticipated data saturation, defined as the point at which additional interviews were unlikely to yield new information relevant to the study objectives. Our sample size was further shaped by logistical constraints related to the COVID-19 pandemic.

Interviews were conducted using an interview guide developed from prior patient-centered qualitive research by our team and refined by the study analyst (ME) through creation of a logic model based on the study protocol [17]. The interview guide, structured around intervention components, incorporated constructs from Social Cognitive Theory, Process Evaluation, Acceptability, and Feasibility [23–25]. The study analyst tabulated existing quantitative and qualitative measures by intervention protocol outcomes as identified by the logic model, which aided members of the research team in identifying themes in the interview guide that could be removed or expanded. The interview guide was finalized based on input of several members of the research team (ME, AL, KS, MC, DE). Patient interviews covered the five components of the comprehensive telehealth intervention, perception of nurse interactions, baseline behavior and changes, knowledge of outside programs, and areas for potential intervention improvement. Staff interviews focused on intervention components that were perceived as beneficial versus less useful, implementation factors, feasibility of fitting the intervention into existing responsibilities, sustainability, interactions with colleagues, and past experiences with interventions. Both interview guides included probing questions to facilitating dialog, if needed (Appendix). Interviews were conducted via telephone and ranged from 13 minutes to 68 minutes, with an average duration of 35 minutes.

### Data analysis

All interviews underwent transcription and directed content analysis [26]. The study analyst (ME) debriefed with qualitative (KS, AL) and content (MC, AL) experts throughout the processes of data collection, codebook development, and data analysis to ensure dependability and credibility [27]. The analyst created process notes, an audit trail document, summary memos, and completed data triangulation between patient and provider data (22). Initial *a priori* codes were derived based on the study protocol and work by Andrews *et al* [17,21]. Additional codes were generated based on feedback from a qualitative and content expert (AL) and inductive code creation (Appendix Table 3). Following the completion of initial coding using ATLAS.ti 9, the data analyst worked with the qualitative expert (KS) to create the structure for the matrix, to explore patients’ general perceptions of “what worked well” and “what did not work” within the intervention components, stratified by telephone encounter engagement [28]. Preliminary themes were presented to the research team, who provided feedback on salience and importance. The analyst used an iterative process of coding, consulting with the research team and recoding, composing memos, and matrix analysis to refine the themes [28]. The Standards for Reporting Qualitative Research were used for organizing and reporting analysis and results (Appendix) [29].

## Results

### Sample characteristics

Interview participants included 19 patients and 8 staff (3 HT nurses, 5 medication managers) who participated in the comprehensive telehealth intervention arm. Patient interviewees were predominately non-Hispanic/Latino Black males, which was reflective of the overall PRACTICE-DM trial population. Table 1 compares patient interviewee demographics to the overall comprehensive telehealth intervention arm population.

**Table 1.** Demographics of semi-structured interview patient participants compared to overall PRACTICE-DM intervention arm. This table provides an overview of the demographics of participants in the qualitative interviews conducted as part of the overall comprehensive telehealth intervention study population. The table compares key characteristics such as sex, race, ethnicity, and other relevant factors between the interviewees and the broader study population, offering insights into the representativeness and diversity of the qualitative sample within the larger context of the study

	Interview Participants (*N* = 19)	PRACTICE-DM Intervention arm (*N* = 101)
**Sex**		
Female	4 (21.1%)	24 (23.8%)
Male	15 (78.9%)	77 (76.2%)
**Race**		
White or Caucasian	9 (47.4%)	25 (24.8%)
Black or African American	10 (52.6%)	68 (67.3%)
Other		8 (7.9%)
**Ethnicity**		
Hispanic / Latino	0	6 (5.9%)
**Site**		
Durham	12 (63.2%)	57 (56.4%)
Richmond	7 (36.8%)	44 (43.6%)
Engagement Level^*^		
**≥ 19 calls**	14 (73.7)	79 (79.0%)
**10–18 calls**	3 (15.8%)	9 (9.0%)
**1–9 calls**	2 (10.5%)	12 (12.0%)
**Average engagement level** (encounters completed of 26 possible encounters)	20	20

* One participant who enrolled in the PRACTICE-DM Intervention arm completed 0 calls; thus, percentages of engagement level are based on 100 participants.

### Findings

**Figure 1.**
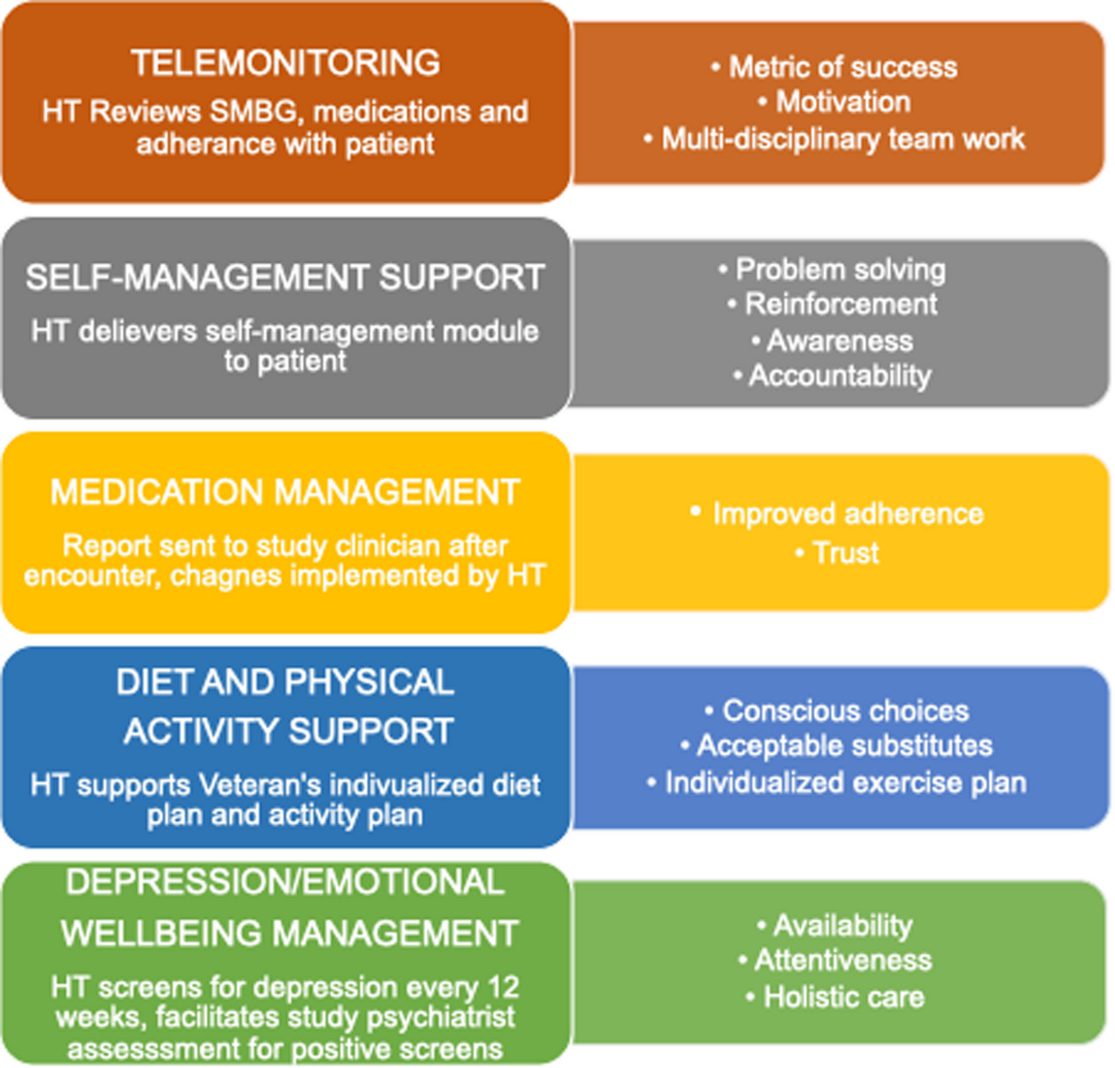
Identified benefits within each telehealth component. This figure highlights common factors that were reported, by patient and staff interviewees, to have worked well within each of the five components of the comprehensive telehealth intervention. HT = home telehealth, SMBG = self-monitoring of blood glucose.

We present aspects of the comprehensive telehealth intervention arm experience from the perspective of patient and staff. Table 1 provides an overview of the demographics of participants in the qualitative interviews conducted as part of the overall comprehensive telehealth intervention study population. Figure 1 highlights identified benefits within each telehealth component. Table 2 summarizes areas of improvement within each telehealth component.

**Table 2.** Identified areas of improvement within each telehealth component perspective. This table presents common areas that were reported, by patient and staff interviewees, to be potential areas of improvement within the individual telehealth components

	Patient	Staff
**Telemonitoring**	Time burden on patients with work constraints Difficulty with data reporting	Delay in data communication Panel size leading to delayed documentation by nurses
**Self-Management support**	Comorbidities such as mental health disorder making it difficult to adhere to program	Difficulty in identifying causes of nonadherence Disconnect between med manager and patients Volume and time limits for module completion Limitations due to comorbidities such as mental health disorder
**Diet and physical activity support**	Work or injury related limitations	Lack of EMR documentation of diet plans
**Medication management**	Frequency of changes Communication with PCP	Patient availability Lack of direct interaction with patient
**Depression support**	May be insufficient for the needs of patients with significant mental health needs	None reported within the scope of staff roles

### Telemonitoring

#### Benefits

Participants described the benefits of the telemonitoring component operating through three factors: *metric of success, motivation, and multidisciplinary teamwork*. First, patients described calls from HT nurses to review records of blood glucoses as encouraging and motivating in their diabetes self-management. Multiple high engagement patients described a sense of accountability fostered by the calls that inspired a subsequently more active role in self-management. “I had to take care of myself and take my medicines so things would come out on a positive end when I talk with the nurse” (high engagement patient, ID#10). Another high engagement patient (ID#9) stated:

“And what also helped was the, the tele-reporting system. That I was able to upload my blood sugar numbers into. […] that gave me structure. And with that structure I was able to make a routine that lasted for longer […] than the duration of the program.”

Second, nurses described a feeling of satisfaction in being able to provide patient participants with a metric of success as it related to glycemic control and noted routine calls kept patients accountable and active in their self-management.

“And I think patients actually, it helps them not just hearing numbers but giving them a percentage. To say, you’re a 100% within goal. I mean that’s what they’re all striving for. The bulk, most people. I mean the ones who care, they’re all striving for a 100%. So, when I say, well last time we talked, you were 10% above goal and now you’re 100% within goal. Then that gives them something really to be proud of. It gives them something to strive for.” (Staff ID#3)

Third, medication managers reported having information related to medication reconciliation, adherence, and blood glucose data in the EHR was useful and having nurses involved offered a level of flexibility and efficiency.

#### Areas for improvement

The major themes regarding potential improvement were *time constraints*, identified by both patients and staff, and *difficulty with data reporting*, identified by patients. Some nurses and medication managers noted that time limits and panel sizes constrained their work. Nurses noted that they found themselves needing to adjust and prioritize patients with severe data abnormalities despite having scheduled time with other patients.

“But also, with home-telehealth, I’m calling patients based on their need. So, if I have a person who has a sky-high blood pressure or some other numbers that are out of whack and they need immediate attention. Then that can sort of delay your time getting to that patient that you have scheduled” (Staff ID #3)

Staff cited this flux in prioritization as a potential cause for delayed documentation being sent to the medication managers. Some patients described the glucometer as burdensome due to testing frequency and device interface complexities. Some patients (moderate to high engagement) expressed difficulty with taking and transmitting data due to traveling and work schedules.

### Self-management support

#### Benefits

Participants described the benefits of the self-management support component operating through four factors: *problem-solving, reinforcement, awareness, and accountability*. Staff were described as an available source for problem-solving, reported across all engagement levels. Staff offered advice, instructions, and solutions, and as a result patients described that they looked forward to speaking with staff about self-management.

“That was—a lot of those ideas came from the nurses, and they were able to give me the tools like my phone, you know, putting information into my phone. I have it stored and I can (…) __see my progress from there.” (high engaged patient, ID#19)

Additionally, some patients (across engagement levels) said their self-management skills were reinforced by the program’s structure, reminders, and routines.

“It just gave me the, it gave me a lot a structure. And the structure it gave me pretty much gave me self, I don’t want to say self-worth. It made me have more self-confidence with dealing with the diabetes.” (low engagement patient, ID#4)

Patients reported gaining greater awareness of their diabetes, self-management behaviors, and the influence of diet on blood glucose. Having to report eating habits and blood sugar readings led to participants having an increase sense of accountability. HT nurses reported patients were very receptive to education and guidance because it was presented with real life applications and with an emphasis on dispelling myths.

“And with this study, we took everyone from step one. And explained to them, hey you may already know this, but we’re just going to review this. And patients in the study were more receptive to that. I think your average Veteran, or your average patient who’s been diabetic for ten, twenty, thirty years, they really don’t want to hear anything because they think they know it all. But with this study, patients were more willing to listen and realized that there were things that they didn’t know.” (Staff ID#3)

A HT nurse (Staff ID#9) identified having flexibility and personalizing self-management support modules with patients worked well:

“Well, you know, the module, I looked at them as kind of like a script. I did not read them to the patient. And I tried to, you know, review the information ahead of time and be able to communicate that to the patient in a way that they could understand and throughout the whole call, not necessarily, okay, now we’re gonna do the module, you know, from the beginning of the call to the end. And sometimes having to go back and reinforce information from before. So, I found the modules very helpful but I just tried not to, you know, read it like a book__.”

A medication manager (Staff ID#6) reflected, “I wish I had somebody that I could count on like that for the rest of my patients. I don’t think I would change anything. I think it was like ideal. The ideal situation.”

#### Areas for improvement

The major themes for areas of potential improvement in the self-management component were related to *patient adherence, time limitations for module completion and interaction between study staff*. A few patients shared that their ability to gain self-management skills was impeded by critical mental health history. One patient with low engagement (ID#5) shared, “…because of my paranoia and fear I just can’t get myself in a regular routine of diabetes management.” In a second suggestion for improvement, a medication manager (Staff ID#4) suggested that support could be improved by speaking directly with patients to gather their perspectives on causes and challenges of nonadherence:

“I would say, just it’s a little more challenging when you’re not directly interacting with patients to pick up on potential flags for nonadherence. Flags with respect to their motivation or lack thereof. Or beliefs when it comes to their diabetes. That could influence or give me more insight to their current state and therefore affect their treatment plan and other ways to focus. Diabetes is one of those clinical areas that’s very dependent on patient lifestyle, patient engagement, and it’s hard. So, it’s sometimes difficult just through the chart to pick up on the patients’ beliefs and motivation looking at the objective data that comes through the chart.”

Finally, we heard from nursing staff, that increasing the flexibility in timing of module completion may increase patient skills, knowledge of self-management skills.

### Diet and physical activity support

#### Benefits

Participants described the benefits of the diet and physical activity support component through three variables: *conscious choices, acceptable substitutes, and individualized exercise plans.* Most patients found the education on diet and physical activity satisfactory, reporting it led to positive behavioral changes including, maintaining diet logs, improved portion control, and increasing physical activity. One high engagement patient (ID#16) explained, “It taught me how to count my carbs and the calories and all that. Which I wasn’t doing before. I wasn’t doing, I was doing a terrible job with it.” Another high engagement patient (ID#22) shared how the intervention encouraged mindfulness around meal composition saying, “It made me more conscious of my diet.” (…) “Because I had to, first off, I had to kind of focus on all right, what is my plate supposed to look like here?.” Patients also reported substituting less healthy foods for better options. One high engagement patient participant mentioned replacing sodas with a beverage with “zero calories, zero sugar.” Another participant described replacing breakfast cereal with eggs. Another high engagement patient (ID#13) reflected:

“Well, where I may have eaten muffins and doughnuts for breakfast, I usually eat a sensible breakfast now. And I don’t snack as much, like my snack between meals is a little healthier. Maybe a piece of fruit or something.”

Multiple participants spoke about implementing portion control, particularly with high glycemic foods like candy, cake, and sodas, and engaged in self-monitoring by logging their food and reading labels to make healthier choices. Participants across various mobility levels reported increased physical activity, with one high engagement patient (ID #18) specifically noting the positive impact of incorporating chair exercise into their physical activity routine. Another high engagement patient (ID#12) stated: “They always motivate me to get out three times a week at least, Thirty minutes.”

Staff echoed the benefits of the intervention component, particularly highlighting the inclusion of a dietician. One medication manager (Staff ID#5) emphasized the holistic approach:

“I appreciated the fact that there was the nutritionist on board, the psychologist on board, it seemed a well-rounded approach to assist the patient. … Was the patient walking, are they doing any type of exercise? … [The HT Nurse] was really great with some real challenging patients about doing food diary with them, or people who had trouble with hypoglycemia… She would follow-up on them weekly for a couple of those people just to really try and get a better handle on what they were doing and how to take care of them in the safest manner.”

Multiple staff members expressed a sense of “teamwork,” with one medication manager (Staff ID#8) stating, “The PRACTICE DM team, the composition of the team was super helpful in just meeting all the Veterans’ needs with the providers, the pharmacists, especially the dietitians, so I liked that.”

#### Areas for improvement

Participants and staff reported limited areas for improvement in the diet and activity component. Staff identified a *lack of diet plans documented in the EHR* as a potential area of improvement in the diet and physical activity component. A medication manager stated there were “growing pains initially because some of the diet stuff wasn’t there” when describing looking for dietary information in the EHR. Two patients reported no change in physical activity since the intervention, one due to an injury that limited their physical activity (high engagement patient), and the other because they were already physically active prior to the intervention (low engagement patient).

### Medication management

#### Benefits

Participants described the benefits of the medication management component operating through two variables: *improved adherence and trust.* Patients found HT-delivered medication management acceptable, and it resulted in improvements in medication adherence and HbA1c levels. One high engagement patient (ID#20) shared:

“… before the study, I was haphazard in taking my medication on a regular basis. And I also found out that I wasn’t taking it properly. There were issues about different ones had to be on an empty stomach, different ones had to be on a, you know with food on my stomach. There were, I shouldn’t have milk with some of them. Things like that that was, it just, it kept me on schedules, and I kept myself out of whack, you know because I just, if I didn’t remember to take medicine in the morning, it would just take it whenever. Now I am very regimented. Make sure I change the schedule of which medicines to take when. It’s really kind of made me more aware and concerned with the program with diabetes and what it means being a diabetic and how to better take care of myself.”

Patients expressed trust in the clinical decisions made by staff, feeling changes were made in their best interest. Multiple patients also shared having frequent contact with nurses ensured timely medication refills.

Intervention staff highlighted the benefits of a collaborative approach to medication management. One medication manager (Staff ID#4) shared, “I think that it really allows clinicians to practice at the highest level of their license.” Another staff member (ID#5) shared that the team-based structure allowed them “to do more for more people.”

#### Areas for improvement

Both patient and staff identified areas for improvement, including *patient availability and interaction, frequent medication changes, and communication with primary care providers (PCPs).* Some high engagement patients expressed concerns that their PCPs were not well-informed about medication changes made during the intervention.

Patient specific barriers, such as anxiety, also limited the medication management component effectiveness for some individuals. One low engagement patient (ID#5) explained:

“For me it didn’t work because of what’s going on with me. I think any other person would have done fine in the setting but with my fears and anxiety and taking medicine it certainly keeps me from, you know, getting diabetes under control or if not under control, better”

HT Nurses, who served as the primary point of contact for patients, faced challenges with timing and scheduling calls due to patient availability, leading to increased time and effort spent. While nurses reported positive reactions to communication with medication managers, one medication manager felt disconnected and expressed a desire for more direct interaction with patients.

### Depression support

#### Benefits

Patients and staff described the benefits of the depression support component to include *availability, attentiveness, and holistic care.* Of the patients who completed interviews, 40% entered the depression protocol after having Personal Health Questionnaire-8 scores ≥ 10, indicating moderate to severe depression. Patients (*N* = 11) frequently praised the availability and reliability of nurses’ calls when asked about emotional support. One high engagement patient (ID#19) shared, “I talked to […] quite a bit throughout the program in […]. And she was a godsend. She was—she kept me going.” Others echoed similar sentiments, using positive phrases such as, “always available,” “always there for me,” and “knowing somebody is there to talk to.” Staff also highlighted the holistic nature of the depression support component. One medication manager (Staff ID#5) described it as “a well-rounded approach (…) to assist the patient.” Another medication manager (Staff ID#7) emphasized how the intervention component improved their understanding of the complex interplay between mental health and diabetes management, stating:

“Just to have a better understanding of what’s going on with the patient and how these things are impacting potentially their ability to do what you would like them to be doing with their medication and checking their sugars.”

#### Areas for improvement

While patient and staff feedback on the Depression Support component of the telehealth intervention was generally positive, some patients with significant mental health needs felt the protocol was insufficient to fully address their challenges. Two patients, both of whom self-identified as having PTSD, expressed that the intervention did not adequately support their diabetes self-management needs. One low engagement patient (ID#5) explained:

“I thought the program could help me get back on track with my diabetes. But this fear is just so prominent that it’s almost like my hands are handcuffed… I get very, very anxious. I sweat, I mean it’s just like—is this gonna hurt me? Is this gonna kill me? Those kinds of thoughts. But also have the thought of trying to do the right thing.”

Similarly, one moderate engagement patient (ID #6) described trauma-related symptoms such as hypervigilance, disrupted sleep cycles, and reliance on sugary caffeinated drinks to manage productivity further complicated self-management.

Staff did not identify specific areas for improvement within the scope of their roles related to the depression support component.

## Discussion

The rising prevalence of T2 DM necessitates innovative care models to address this public health challenge and its impact on patients and healthcare systems. Telehealth, with its capacity to provide frequent monitoring and personalized support, offers a promising solution. This qualitative analysis highlights patient and staff perspectives on the five components of a comprehensive telehealth intervention and how they perceived these components to support improved T2 DM management and areas of potential intervention refinement in the context of PPDM. This work centers on the participants experiences and perspectives with the aim of generating hypotheses for future research.

### Key contributions of intervention components

Participants reflected on how specific components of the intervention help support their diabetes care. Telemonitoring was perceived to promote accountability and motivation by providing structure and consistent feedback. Self-management support was described as offering useful problem-solving strategies, reinforcing routines, and increasing patient awareness of diabetes behaviors while empowering patients to take an active role in managing their diabetes. The medication management component was viewed as supportive in helping patients adhere to treatment regiments and build trust in clinical recommendations. Diet and physical activity support were linked to conscious dietary changes, such as improved portion control, healthier substitutions, and individualized exercise plans. Depression support was highlighted for addressing emotional well-being alongside physical health. These insights reflect the ways in which a comprehensive telehealth intervention may meet the diverse and intersecting needs of patients with PDM. These perspectives suggest that the perceived value of the intervention stemmed not only from the content delivered but also from how it was delivered. For high-risk populations like those with PPDM, these relational and structural features may be especially critical to intervention success.

### Opportunities for refinement

While the intervention was overall well-accepted, opportunities for refinement were identified that may enhance both patient engagement and the overall effectiveness of the comprehensive telehealth approach. Within the telemonitoring component, challenges such as time constraints for staff, and difficulties with glucose testing frequency, and device interfaces for patients suggest the need for innovations that reduce patient burden and optimize staff efficiency. Tailoring self-management support to better accommodate individual patient circumstances, such as integrating self-management strategies across multiple chronic conditions or applying trauma-informed principles to support patients with PTSD or anxiety, may improve patient engagement and increase the component’s effectiveness. The medication management component highlighted the need for improved care coordination between interventionists and PCPS to ensure continuity. Within the depression support component, there was a need for additional strategies to support patients with more severe mental health challenges. Future work addressing these areas may amplify the care approach and improve outcomes for these patients. Building on these findings, our team is currently conducting a secondary qualitative analysis of interview transcripts to better understand implementation barriers and facilitators, with the goal of identifying opportunities to support translation of evidence-based comprehensive telehealth approaches in real-world care settings.

### Findings in the context of prior research

This study builds on and extends prior research on the use of telehealth interventions for chronic disease management. Previous qualitative studies have identified benefits of technology- assisted telehealth interventions including improved self-management skills [17,19,30], and an acceptable means to provide diabetes education [31] that results in improved patient confidence and lifestyle habits and promotes adherence to treatment plans [32]. However, few studies have explored how both patients and staff experience multiple integrated components of a comprehensive intervention in a real-world setting for the management of PPDM. Our findings contribute to the literature by offering a nuanced exploration of how individual components were perceived to support diabetes care, what features were viewed as especially impactful and where improvements may be needed. In doing so, this study provides a practical foundation for refining the design and delivery of comprehensive telehealth programs. Importantly, our results also align with and reinforce prior work emphasizing the value of behavioral health integration in diabetes care. By addressing emotional well-being alongside physical health, telehealth intervention with behavioral therapy can support patients in overcoming psychosocial barriers to effective diabetes self-management [33]. Our findings are consistent with literature demonstrating certain forms of telemonitoring in diabetes management can viewed as burdensome by patients [34] and increase workload for staff [35]. These insights underscore the importance of balancing comprehensiveness with feasibility and support the value of multifaceted, team-based approaches to telehealth.

### Strengths and limitations

A notable strength of this study is its robust methodology, which included the use of a theoretical framework to guide design and analysis. Additionally, including perspectives from both patients and staff enabled a comprehensive understanding of how the intervention was experienced and where improvements might be made. We acknowledge the predominantly male, Veteran sample, which may limit transferability to non-Veteran populations and those that are not predominately male. The absence of Hispanic/Latino study participants may limit insights into cultural influences on the intervention engagement and effectiveness.

## Conclusion

This study underscores the importance of engaging both patients and staff to understand how a comprehensive telehealth intervention experienced in real-world settings to address the complex needs of patients with PPDM. Participants described how different variables, such as structured feedback, personalized education, and emotional support, contributed to their diabetes care and identified areas of improvement. These perspectives offer practical insights that can inform future program refinement and generate hypothesis for further research. Comprehensive telehealth models may help bridge gaps in traditional clinic-based care by addressing both medical and behavioral health needs, particularly for patients with PPDM. As healthcare systems seek scalable, patient-centered approaches to chronic disease management, leveraging patient and staff feedback will be critical to maximizing impact and improving outcomes across diverse care settings.

## Supporting information

10.1017/cts.2025.10082.sm001 German et al. supplementary material German et al. supplementary material

## Supplementary material

The supplementary material for this article can be found at https://doi.org/10.1017/cts.2025.10082.
