# Supplementary material for: Perceptions of a comprehensive telehealth intervention in patients with persistently poor type 2 diabetes control
Source: J Clin Transl Sci. 2025 Jun 25;9(1):e153. doi: 10.1017/cts.2025.10082 (PMC12392345; doi:10.1017/cts.2025.10082)
Supplement: German et al. supplementary material [file S2059866125100824sup001.pdf]

Supplemental Material

Sample PRACTICE-DM Self-Management Support Module

Qualitative Interview Guide for Patient Participants (Appendix Table 1)

Qualitative Interview Guide for PRACTICE-DM for Staff Participants (Appendix Table 2)

Data Analysis Code Book (Appendix Table 3)

Standards for Reporting Qualitative Research (SRQR)

APPENDIX D: SAMPLE PRACTICE-DM SELF-MANAGEMENT SUPPORT MODULE

PRACTICE-DM MODULES
Blood Glucose Monitoring Module (Encounter 2)

Study ID: __________ **Call Duration (minutes): ___________**

Number of call attempts: ________________ **Date Completed: ___________**

Notes (optional): ____

____

____

____

Call components (check each as completed):

	**Encounter 2**<br>*4 wks post- enrollment*	**Completed?**
**Tele-monitoring**	SMBG data review/general adherence assessment	
**Self-Mgmt. Support**	Module: Self-monitoring blood glucose	
**Diet/activity Support**	Notify dietitian to contact pt	
**Diabetes Med. Mgmt.**	Medication reconciliation	
**Depression Support**	Confirm psych. eval arranged (if PHQ8 was +)	
**Follow-up**	*During next wk*:<br>• Call to relay med. changes<br>• Study dietitian call to develop diet plan	
**CPRS Tracking**	Enter notes in CPRS	
**Study Tracking**	Open the study database and complete the survey to complete study documentation.	

2) Self-monitoring of blood glucose (1)

2.00 As we discussed at the beginning of this project, blood sugar monitoring is an important part of self-managing your diabetes. The purpose of this module is to make sure you have everything you need to correctly monitor your blood sugars. It is possible that you have heard some of this information before. By the end of this phone call, our goal will be to make sure you have a clear plan for monitoring your blood sugar.

Blood glucose monitoring is a way to measure blood sugar levels anytime on a small portable meter. Monitoring is done by pricking a finger and then putting a small drop of blood on a testing strip (this is also called "a fingerstick"). The strip is read by the meter and then displays a blood sugar number on a digital display. Checking your blood sugar this way allows you to respond to high or low blood sugar levels and quickly take any needed actions. *(go to 2.01)*

2.01 Are you using a blood glucose (sugar) meter?

_____ Yes *(go to 2.04)*

_____ No *(go to 2.02)*
_____ DK *(go to 2.06)*
_____ Refused *(go to 2.06)*

2.02 What is the main reason you are not using a blood glucose (sugar) meter?
_____ Finances *(go to 2.03)*
_____ Don't know where/how to get one *(go to 2.03)*
_____ Unable to use one *(go to 2.05)*
_____ Don't know how to use one *(go to 2.05)*
_____ Dislike using one *(go to 2.06)*
_____ Don't want one *(go to 2.06)*
_____ DK *(go to 2.06)*
_____ Refused *(go to 2.06)*

2.03 Blood sugar meters and supplies are often available for diabetes patients through their primary provider's clinic, especially for those who use insulin. Our project staff can help coordinate getting a blood sugar meter for you, and you can come in to your primary provider's office to receive training. Can we arrange for you to get a meter and supplies and arrange a training session at your primary provider's clinic?
_____ Yes *(go to 2.06)*
_____ No *(go to 2.06)*

2.04 Are you having any problems using your meter?
_____ Yes *(Assess type problem; provide teaching help if able)* *(go to 2.05)*
_____ No *(go to 2.06)*
_____ DK *(go to 2.06)*
_____ Refused *(go to 2.06)*

2.05 All meters should come with instructions. In addition there is a 1- 800 number on the back that you can call for help with your exact meter type. Make sure you are using the correct strips for the model you own (the code on the bottle of strips should match the code on the meter). If you are having a lot of trouble using your meter, we can also arrange training with your primary care provider's office. Would you like us to arrange a training session at your primary care provider's office?
_____ Yes *(go to 2.06)*
_____ No *(go to 2.06)*

2.06 How often are you told to monitor your blood sugars (or do fingersticks)?
(allow participant to respond and then check any that apply)
_____ Every day *(go to 2.07)*
_____ 1st thing in the morning before I eat anything *(go to 2.07)*
_____ Before meals *(go to 2.07)*
_____ Before and after meals *(go to 2.07)*
_____ Morning and evening *(go to 2.07)*
_____ Only "if I feel bad" *(go to 2.07)*
_____ Other: *(specify)*:____________________________ *(go to 2.07)*
_____ Don't monitor *(go to 2.14)*
_____ DK *(go to 2.07)*
_____ Refused *(go to 2.07)*

2.07 Trying to test blood sugar on a regular schedule can be hard. What, if anything, keeps you from doing fingersticks on a regular schedule?
(allow participant to respond and then check any that apply)
_____ Discomfort involved *(go to 2.08)*
_____ Remembering to do it *(go to 2.09)*
_____ Gathering all the supplies *(go to 2.09)*
_____ Making time to do it *(go to 2.09)*

_____ Need help to do it *(go to 2.10)*
_____ Cost of supplies *(go to 2.12)*
_____ Other *(specify)*:___________________________ *(go to 2.08)*
_____ None of above (test on a regular schedule) *(go to 2.14)*
_____ DK *(go to 2.14)*
_____ Refused *(go to 2.14)*

2.08 It is important not to skip fingersticks. To lessen discomfort, make sure you to use the side of your fingertip and not the more sensitive tip. Use different fingers to avoid bruising. Also, be sure to wash your hands in <u>warm</u> soapy water, dry them well, and then shake your hand down at your side for a few seconds to help improve the blood flow to your fingertip.
(go to 2.09 if memory, gathering supplies or making time answered)
(go to 2.10 if needing help answered)
(go to 2.12 if cost is a problem)
(go to 2.14 if no other barriers identified)

2.09 Remembering to do fingersticks can be hard sometimes. Try to make testing part of your morning routine – before you start fixing your breakfast or taking your other medicines. Have all your supplies already grouped in a special spot so you are ready to go. Also, keeping a log/calendar of your results out in plain sight may also help you to remember.
(go to 2.10 if needing help answered)
(go to 2.12 if cost is a problem)
(go to 2.14 if no other barriers identified)

2.10 What type of help do you need to perform fingersticks? *(all responses go to 2.11)*
_____ Physical (unable to manipulate meter or supplies)
_____ Visual
_____ Reminders (due to poor memory)
_____ Other *(specify)*: ______________________
_____ DK
_____ Refused

2.11 If you need help with fingersticks, is someone usually available to help on a daily basis?
_____ Yes *(go to 2.14)*
_____ No *(go to 2.13)*
_____ DK *(go to 2.13)*
_____ Refused *(go to 2.14)*

2.12 Testing supplies can be expensive – especially if you are testing often, but your providers may be able to help you get the testing supplies you need *[Continue w/ 2.12a if meter/supplies were not ordered under 2.03 above.]*

2.12a Our project staff can help coordinate getting a blood sugar meter for you, and you can come in to your primary provider's office to receive training. Can we arrange for you to get a meter and supplies and arrange a training session at your primary provider's clinic?
_____ Yes *(go to 2.14)*
_____ No *(go to 2.14)*

2.13 If fingersticks are difficult for you to do alone, consider asking a family member or reliable neighbor to help you. If this is not possible, please talk to your VA social worker, provider, or diabetes nurse to see if you qualify for any assistance programs in your area.
_____ Yes *(provide numbers from county resource list; then go to 2.14)*
_____ No *(go to 2.14)*
_____ DK *(go to 2.14)*
_____ Refused *(go to 2.14)*

2.14 How do you keep track of your blood sugar results?

_____ On a calendar *(go to 2.15)*
_____ Keep a log, notebook, chart or list *(go to 2.15)*
_____ Download meter to the computer *(go to 2.15)*
_____ Just keep sugars on meter *(go to 2.15)*
_____ Other: *(specify):*____________________ *(go to 2.15)*
_____ DK *(go to 2.15)*
_____ Refused *(go to 2.15)*

2.15 *(go to 2.16)*

2.16 We recommend that you track your blood sugar result in a reliable way so that you can transmit them regularly using your Home Telehealth equipment. The most reliable way to log your sugars is to keep a blood sugar log, although just keeping the sugars on your meter may be acceptable if you are able to upload your sugars daily. *(go to 2.17)*

2.17 How often you should check your sugars depends on what kind of medications you take for your diabetes. Can you confirm what kind of medications you are taking for your diabetes?

_____ 2 or more insulin shots with or without pills *(go to 2.18)*
_____ One insulin shot with or without pills *(go to 2.19)*
_____ Pills alone, no insulin *(go to 2.19)*
_____ Other:
*(specify):*____________________ *(go to 2.19)*

2.18 Because you take two or more insulin shots daily, it is recommended that you check your blood sugar four times daily - before each meal and at bedtime. Please transmit your sugars regularly as directed. *(go to 1.20)*

2.19 Because you take one or fewer insulin shots daily, it is recommended that you check your blood sugar two times daily – in the morning on an empty stomach and sometime later in the day before lunch, dinner, or bedtime. It would be best if you vary the time you check your second sugar. It is possible that we may ask you to increase the number of sugars you check each day in the future. Please upload your sugars to the Home Telehealth program regularly as directed. *(go to 2.20)*

2.20 Do you have questions about anything we have covered as part of this module?

Note to HT provider: Enter CPRS note and request co-signature of PRACTICE-DM program PharmD/Program Provider. Document any pertinent information in CPRS note addendums and communicate with patient as necessary. Arrange follow-up call as necessary to relay pertinent information back to the patient within 2 business days. Next module call to be completed in 10-14 days.

Follow up call attempts: ______________

Follow up call completed date: ______________

Follow up call duration: ______________

Notes (optional):

____

____

Table 1: Practice DM Patient Interview Guide
• Overall, how would you describe your emotional well-being during the Practice-DM Program?<br>• How did your emotional wellbeing affect how you cared for yourself and your diabetes, if at all?<br>• What were ways, if any, you received emotional support for living with diabetes from the Practice-DM program?<br>• What made you want to talk to the nurse? If you were motivated to talk to the Practice-DM nurse, what led to that?<br>• What, if anything, made you not want to talk to the PRACTICE-DM nurse about your self-management?<br>• How did talking with the nurse influence how you cared for yourself and your diabetes?<br>• Please describe an instance where you found the nurse to be helpful for managing your diabetes? Or unhelpful? Any suggestions about how we could improve the time with the nurse? Are there other things you could discuss that would be helpful?<br>• How did you feel about having your insulin or other medications adjusted by phone? What worked well? What did not work?<br>• Was there anything difficult about measuring your blood sugars? If so, what? What would make it easier for you to check and transmit your home blood sugar readings?<br>• How did it affect your diet? the way you take your medications? otherwise influence your life?<br>• What do you see as the benefit, if any, of at-home blood sugar readings?<br>• What is your opinion about the extent to which that is helpful in overall regular VA care?<br>• What was helpful or not helpful about the information the nurse shared with you? Did it effect your day-to-day behavior?<br>• How did this information affect the way you monitor your sugar? take your insulin? follow recommendations about diet and physical activity?<br>• What did you like or not like about the diet and physical activity plan that the nurse created with you?<br>• What, if any, other programs have you used while you have participated in PRACTICE-DM?<br>• How have these programs influenced how you manage your diabetes and overall health?<br>• How did you find out about these programs?<br>• What changes would you make to the program? What was missing? What would you remove? What other individuals, if any, would you want to be included in this?<br>• Is there anything that you would like to mention that I didn't ask about?

Table 2: Practice DM Staff interview guide

Nurse

- What was useful and what was less useful? What worked well? What didn't work well?
  - Do you have ideas about how it could be improved?
- What, if anything, was challenging about fitting the Practice-DM duties into your usual work duties? What worked well? What didn't work well? (Considering encounter calls, modules, communication with med manager, and relaying med adjustments)
- What worked well with phone interactions with patients with respect to blood sugar monitoring? What would you change? What did not work?
- What aspects of the process of relaying medication adjustment recommendations worked well? What would you change? What did not work?
- How did you prepare for the first phone interaction with a patient?
- How did you prepare for follow-up phone interactions with a patient?
- How did patients react when you talked to them about the idea of the PRACTICE-DM program?
  - What questions did they ask?
  - What were common reasons patients participated?
  - For patients who declined to participate, what were some typical reasons?
- In general, what was it like to interact with the medication manager? What worked well? What would you like to improve about the process?
- What barriers might Practice-DM encounter in routine clinical practice?
- What things would help Practice-DM work well in a routine clinical practice?
- What, if any, other initiatives at your facility are you aware of for patients with diabetes?
  - How do these programs impact patients with diabetes?
- Is there anything that you would like to mention that I didn't ask about?

Medication Manager

- Tell me about your impression of the PRACTICE-DM program. What did you like about it and what did you not like, or find less useful?
- Please describe you r typical workday and duties
- How did the PRACTICE-DM program as a whole fit into your usual work duties?
- What, if anything, was challenging about fitting the Practice-DM duties into your usual work duties?
- In general, what was it like to interact with the program nurse?
  - How did the process of receiving data from the nurse and giving recommendations for medication changes work?
  - How available was the nurse for dialog with you when necessary?
- How would you change the PRACTICE-DM program with respect to interacting with the program nurse?
- Do you think that PRACTICE-DM can become part of routine clinical practice in VA? Why or why not?
- What challenges can you envision to using the PRACTICE-DM program in clinical practice at your VA?
  - How might these challenges be overcome?
  - What would help sustain the use of PRACTICE-DM in clinical practice?
- Can you discuss examples of patient care interventions that have been used successfully in practice at your VA? What worked well about these interventions?
- Can you discuss how you learn about novel patient interventions at your VA?
- What, if any, other initiatives at your facility are you aware of for patients with diabetes?
- How do these programs impact patients with diabetes?
- Is there anything that you would like to mention that I didn't ask about?

Table 3. Codebook

Code	Brief definition	Code Origin
Intervention Components and Structure		
Depression/Emotional wellbeing	Content around participants emotional wellbeing, and support received from the program for emotional wellbeing and/or depression. May capture instances from outside of the program (i.e., support received from relatives and friends). Also used to capture provider mentions of depression or emotional wellbeing of patients and intervention	A priori
Diet/Activity Support	content related to diet and physical activity, the diet plan and physical activity plan, (behavior of the participants and provider descriptions)	
Medication Management	content about adjustment and management of medications for the participants	
Self-Management Support	Content related to behavioral strategies and skills for self-management of diabetes. Support the patient received for self-management or support they felt they obtained as participating in the program. Includes provider mentions of these strategies and the modules.	
Telemonitoring	Content related to use of the HT-issued equipment, and collection of SMBG data. Also includes the transfer of the blood glucose data from the patient to the nurse during the intervention. Includes calls from HT nurses to patients when patients do not upload data or calls for when SMBG numbers are low or high. Includes patient thoughts on and reactions to the telemonitoring. Can also include nurse activities on the study side such as chart review, whether before an enrollment call, or a regular study encounter	
Nurse Role	How the patient describes the role of the nurse in the study, and in their diabetes management	Data derived
Patient Role	How the patient characterizes and views their role in the study/their diabetes care. (Related to motivation, self-efficacy, responsibility, and accountability). Also, how the provider characterizes the patient role.	
Nurse Interactions– PROVIDER	Content about nurse interactions with the medication manager, medication manager interactions with the nurse, as well as attitudes around this	Added for Provider Analysis
Nurse Interactions – PATIENT	content about patient interaction/engagement with the nurse	A priori

What worked well	Used to track aspects of the intervention that worked well, were well-liked, or effective. Look for descriptions such as "helpful", "great", "I liked it" etc. Apply when patients describe improvements to their health or self-management that they attribute to P-DM, or to activities P-DM required. Apply when providers describe aspects of the intervention that patients liked as well, or that worked well for them as providers.	Matrix Code
What did not work	Used to track aspects of the intervention that either worked poorly or were said to need improvement or changes. Look for descriptions like, "awkward", "disconnect", etc. Used to track suggested improvements. May also track context around why something did not work.	
Future P-DM Barriers	Apply this code when providers describe things that would hold back/make it difficult for P-DM to be successful in future practice. (Inside VA, as opposed to on the patient side)	Added for Provider Analysis
Future P-DM Facilitators	Apply this code when providers speak about what things would help make P-DM successful/possible in future practice. (Inside VA, as opposed to on the patient side)	
Patient factors/ behaviors		
Accountability	Mentions of being held accountable, accountability. Relating to study activities and activities of diabetes self-care This can include being held accountable by others (i.e., stuff staff) or by oneself.	Data derived
Self-efficacy	"a person's confidence in their ability to perform a behavior that leads to an outcome." (Glanz et al., 2015) The self-management component of P-DM intervened on Self-Efficacy (and Knowledge). This code covers instances where participants describe their confidence in their ability to manage their diabetes.	A priori
Motivation	Content around the motivation of the participant	Data derived
Structure	Mentions of "structure" and how the study provided "structure", guidance, a foundation, routine, etc…. Mentions of "schedule" as well, as it relates to eating, measuring blood sugar, taking insulin and medications, and physical activity.	
Memory	Any mentions of memory, and memory problems. This may include how memory and memory problems impacted diabetes self-management.	

**Program Impact**		
General Practice-DM Impact	Use to capture any time a participant discusses how any aspect of the intervention impacted their behavior, choices, lifestyle, or thinking	A priori
Health Care Practice-DM Impact	interactions with the healthcare system that Practice DM helped with	
Family/Friends P-DM Impact	interactions with family/friends as a result of Practice-DM.	
Modifications	practices providers describe engaging in in order to accommodate P-DM/to succeed at implementing P-DM.	Added for Provider Analysis
Challenges	Apply this code when providers (HT Nurses and Med Managers) answer about the challenges of implementing P-DM/fitting the Practice DM duties into their usual work duties. This can include things like patient availability.	
Changes	Any suggested changes that participants or providers say they would make to the program.	
**Factors beyond Program**		
Primary Care	Mentions of Primary Care and related content, usually about the relationship of the study to the patient's Primary Care and any communication between the study and primary care	Data derived
Outside Programs	Content about programs other than Practice-DM that participants were involved in or heard about, and who they heard it from.	A priori
Comparison	Use this to capture instances of providers comparing Practice-DM either to regular clinical care or to regular Home Telehealth care.	Added for Provider Analysis

Standards for Reporting Qualitative Research (SRQR)*

http://www.equator-network.org/reporting-guidelines/srqr/

Page/line no(s).

Title and abstract

**Title** - Concise description of the nature and topic of the study Identifying the study as qualitative or indicating the approach (e.g., ethnography, grounded theory) or data collection methods (e.g., interview, focus group) is recommended	1
**Abstract** - Summary of key elements of the study using the abstract format of the intended publication; typically includes background, purpose, methods, results, and conclusions	2

Introduction

**Problem formulation** - Description and significance of the problem/phenomenon studied; review of relevant theory and empirical work; problem statement	3-4
**Purpose or research questio**n - Purpose of the study and specific objectives or questions	4

Methods

**Qualitative approach and research paradigm** - Qualitative approach (e.g., ethnography, grounded theory, case study, phenomenology, narrative research) and guiding theory if appropriate; identifying the research paradigm (e.g., postpositivist, constructivist/ interpretivist) is also recommended; rationale**	4-7
**Researcher characteristics and reflexivity** - Researchers' characteristics that may influence the research, including personal attributes, qualifications/experience, relationship with participants, assumptions, and/or presuppositions; potential or actual interaction between researchers' characteristics and the research questions, approach, methods, results, and/or transferability	4-7
**Context** - Setting/site and salient contextual factors; rationale**	4-7
**Sampling strategy** - How and why research participants, documents, or events were selected; criteria for deciding when no further sampling was necessary (e.g., sampling saturation); rationale**	4-7
**Ethical issues pertaining to human subjects** - Documentation of approval by an appropriate ethics review board and participant consent, or explanation for lack thereof; other confidentiality and data security issues	4
**Data collection methods** - Types of data collected; details of data collection procedures including (as appropriate) start and stop dates of data collection and analysis, iterative process, triangulation of sources/methods, and modification of procedures in response to evolving study findings; rationale**	4-7
**Data collection instruments and technologies** - Description of instruments (e.g., interview guides, questionnaires) and devices (e.g., audio recorders) used for data collection; if/how the instrument(s) changed over the course of the study	4-7

**Units of study** - Number and relevant characteristics of participants, documents, or events included in the study; level of participation (could be reported in results)	4-7, table 1
**Data processing** - Methods for processing data prior to and during analysis, including transcription, data entry, data management and security, verification of data integrity, data coding, and anonymization/de-identification of excerpts	4-7
**Data analysis** - Process by which inferences, themes, etc., were identified and developed, including the researchers involved in data analysis; usually references a specific paradigm or approach; rationale**	4-7
**Techniques to enhance trustworthiness** - Techniques to enhance trustworthiness and credibility of data analysis (e.g., member checking, audit trail, triangulation); rationale**	4-7

Results/findings

**Synthesis and interpretation** - Main findings (e.g., interpretations, inferences, and themes); might include development of a theory or model, or integration with prior research or theory	**7-19**
**Links to empirical data** - Evidence (e.g., quotes, field notes, text excerpts, photographs) to substantiate analytic findings	**7-19**

Discussion

**Integration with prior work, implications, transferability, and contribution(s) to the field -** Short summary of main findings; explanation of how findings and conclusions connect to, support, elaborate on, or challenge conclusions of earlier scholarship; discussion of scope of application/generalizability; identification of unique contribution(s) to scholarship in a discipline or field	19-22
**Limitations** - Trustworthiness and limitations of findings	21-22

Other

**Conflicts of interest** - Potential sources of influence or perceived influence on study conduct and conclusions; how these were managed	1
**Funding** - Sources of funding and other support; role of funders in data collection, interpretation, and reporting	23

*The authors created the SRQR by searching the literature to identify guidelines, reporting standards, and critical appraisal criteria for qualitative research; reviewing the reference lists of retrieved sources; and contacting experts to gain feedback. The SRQR aims to improve the transparency of all aspects of qualitative research by providing clear standards for reporting qualitative research.

**The rationale should briefly discuss the justification for choosing that theory, approach, method, or technique rather than other options available, the assumptions and limitations implicit in those choices, and how those choices influence study conclusions and transferability. As appropriate, the rationale for several items might be discussed together.

Reference:

O'Brien BC, Harris IB, Beckman TJ, Reed DA, Cook DA. **Standards for reporting qualitative research: a synthesis of recommendations.** *Academic Medicine*, Vol. 89, No. 9 / Sept 2014
DOI: 10.1097/ACM.0000000000000388
